# The genome sequence of the Brown Rustic, *Charanyca ferruginea *(Esper, 1785)

**DOI:** 10.12688/wellcomeopenres.20137.1

**Published:** 2023-11-23

**Authors:** Douglas Boyes, Finley Hutchinson, University of Oxford and Wytham Woods Genome Acquisition Lab, Darwin Tree of Life Barcoding collective, Wellcome Sanger Institute Tree of Life programme, Wellcome Sanger Institute Scientific Operations: DNA Pipelines collective, Tree of Life Core Informatics collective, Darwin Tree of Life Consortium

**Affiliations:** 1 UK Centre for Ecology & Hydrology, Wallingford, England, UK; 2 University of Exeter, Penryn, England, UK

**Keywords:** Charanyca ferruginea, Brown Rustic, genome sequence, chromosomal, Lepidoptera

## Abstract

We present a genome assembly from an individual female *Charanyca ferruginea* (the Brown Rustic; Arthropoda; Insecta; Lepidoptera; Noctuidae). The genome sequence is 854.6 megabases in span. Most of the assembly is scaffolded into 31 chromosomal pseudomolecules, including the Z and W sex chromosomes. The mitochondrial genome has also been assembled and is 15.36 kilobases in length. Gene annotation of this assembly on Ensembl identified 23,126 protein coding genes.

## Species taxonomy

Eukaryota; Metazoa; Eumetazoa; Bilateria; Protostomia; Ecdysozoa; Panarthropoda; Arthropoda; Mandibulata; Pancrustacea; Hexapoda; Insecta; Dicondylia; Pterygota; Neoptera; Endopterygota; Amphiesmenoptera; Lepidoptera; Glossata; Neolepidoptera; Heteroneura; Ditrysia; Obtectomera; Noctuoidea; Noctuidae; Xyleninae; *Charanyca*; *Charanyca ferruginea* (Schiffermüller, 1775) (NCBI:txid987475).

## Background

The Brown Rustic *Charanyca ferruginea* (Esper, 1785) (syn. *Rusina ferruginea*) is a medium-sized moth of the family Noctuidae. Despite its drab appearance it is usually fairly distinctive, especially the males which have five or six whitish spots along the leading edge of the relatively broad forewings, a faint dark cross-band halfway down the forewing, and a few lighter crossbands. The species is sexually dimorphic: the females lack the strongly feathered antennae of the males, are smaller, have narrower forewings and are generally more obscurely marked. This occasionally leads to confusion with dark forms of the Square-spot Rustic *Xestia xanthographa* (Denis & Schiffermüller, 1775), although that species lacks the whitish spots along the forewing edge ( Waring *et al.*, 2017).

*C. ferruginea* is widespread across much of Europe, and is a common species across most of Britain, occurring in a range of habitats, while in Ireland and the Channel Islands it is similarly widespread but more localised. Despite this, the species’ abundance has shown a significant decrease in recent years, dropping by 45% between 1970 and 2016 according to data collected by Rothamsted Research ( Randle *et al.*, 2019). The larvae occur on various herbaceous plants from August to May, feeding at night and overwintering almost fully-grown before pupating in a cocoon underground in Spring ( Henwood *et al.*, 2020). Larvae are equally drab, being yellowish- or orangey-brown and increasingly pale along the abdominal segments. A white dorsal line along the thorax is reduced to dots or dashes, or entirely absent, along the abdomen, and there is a faint subdorsal line on each side of the body. Spiracles are black, separating it from similar species *Dypterygia scabriuscula* (Linnaeus, 1758) and *Hyppa rectilinea* (Esper, 1796) which have white or orange spiracles ( Henwood *et al.*, 2020). Adults fly in a single generation in May or June-July, and come to light, sugar and flowers ( Waring *et al.*, 2017).

We present a chromosomally complete genome sequence for *Charanyca ferruginea*, based on one female specimen from Wytham Woods, Oxfordshire, as part of the Darwin Tree of Life Project. This project is a collaborative effort to sequence all named eukaryotic species in the Atlantic Archipelago of Britain and Ireland.

## Genome sequence report

**Figure 1.**
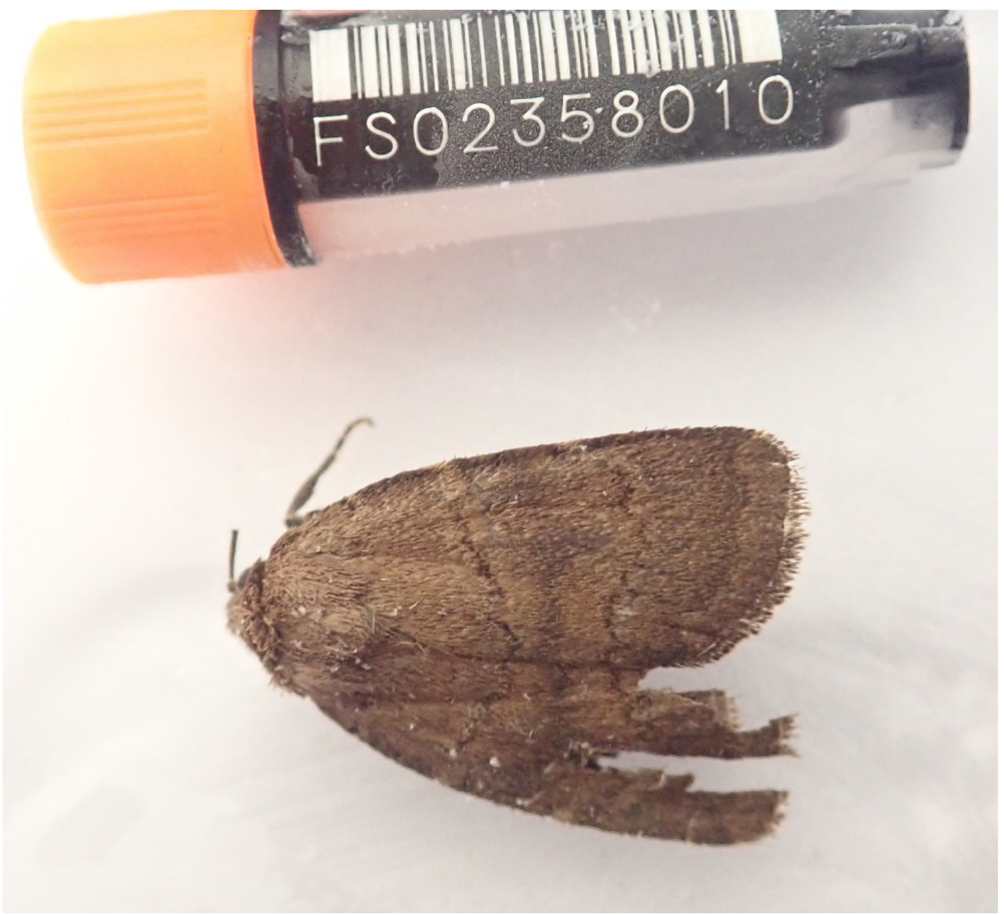
Photograph of the *Charanyca ferruginea* (ilChaFerr1) specimen used for genome sequencing.

The genome was sequenced from one female *Charanyca ferruginea* ( Figure 1) collected from Wytham Woods, Oxfordshire, UK (51.77, –1.34). A total of 46-fold coverage in Pacific Biosciences single-molecule HiFi long reads was generated. Primary assembly contigs were scaffolded with chromosome conformation Hi-C data. Manual assembly curation corrected 99 missing joins or mis-joins and removed 4 haplotypic duplications, increasing the assembly length by 1.7% and reducing the scaffold number by 42.31%, and increasing the scaffold N50 by 1.09%.

**Figure 2.**
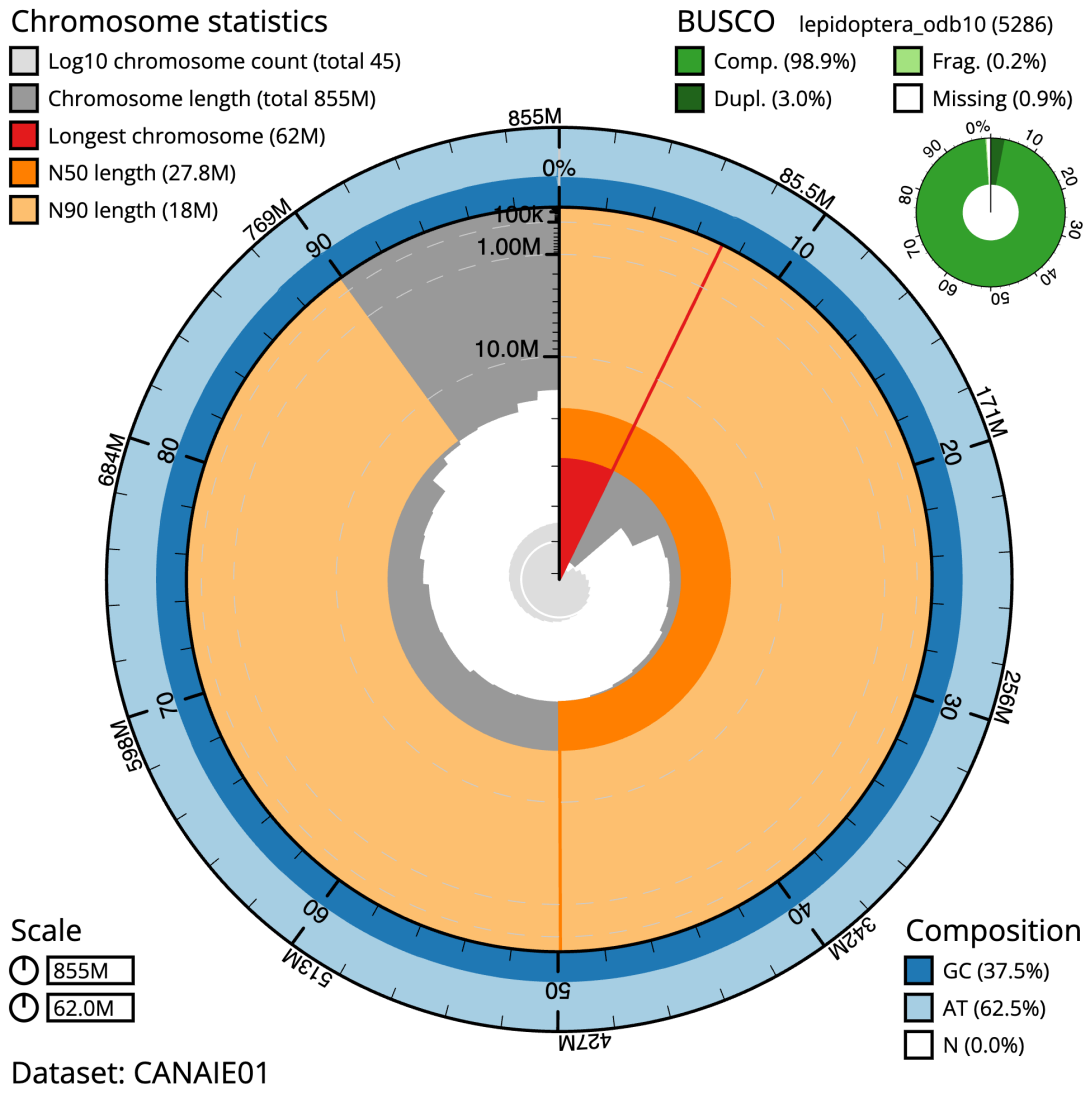
Genome assembly of *Charanyca ferruginea*, ilChaFerr1.1: metrics. The BlobToolKit Snailplot shows N50 metrics and BUSCO gene completeness. The main plot is divided into 1,000 size-ordered bins around the circumference with each bin representing 0.1% of the 854,663,949 bp assembly. The distribution of scaffold lengths is shown in dark grey with the plot radius scaled to the longest scaffold present in the assembly (61,979,575 bp, shown in red). Orange and pale-orange arcs show the N50 and N90 scaffold lengths (27,761,565 and 18,037,562 bp), respectively. The pale grey spiral shows the cumulative scaffold count on a log scale with white scale lines showing successive orders of magnitude. The blue and pale-blue area around the outside of the plot shows the distribution of GC, AT and N percentages in the same bins as the inner plot. A summary of complete, fragmented, duplicated and missing BUSCO genes in the lepidoptera_odb10 set is shown in the top right. An interactive version of this figure is available at https://blobtoolkit.genomehubs.org/view/Charanyca%20ferruginea/dataset/CANAIE01/snail.

**Figure 3.**
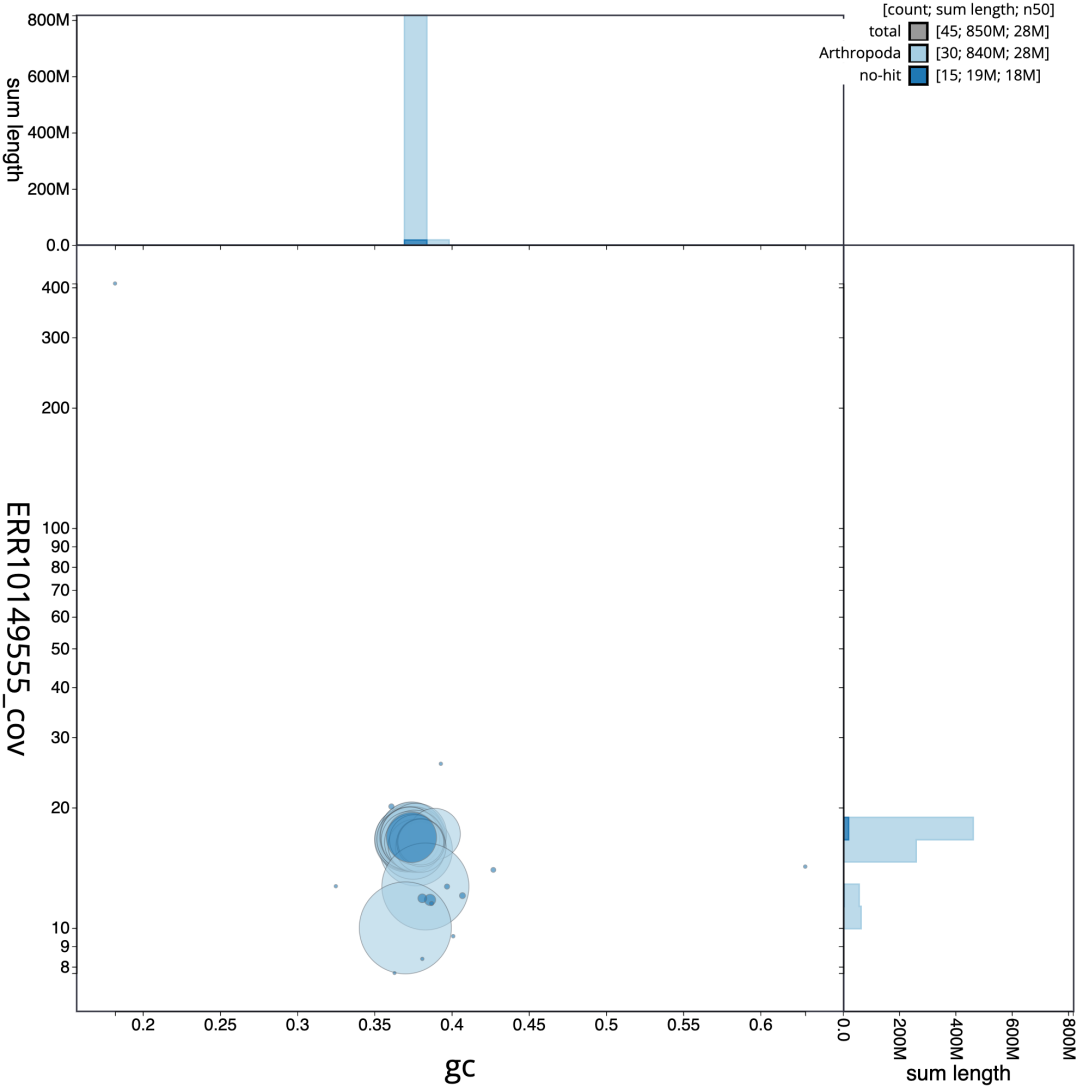
Genome assembly of *Charanyca ferruginea*, ilChaFerr1.1: BlobToolKit GC-coverage plot. Scaffolds are coloured by phylum. Circles are sized in proportion to scaffold length. Histograms show the distribution of scaffold length sum along each axis. An interactive version of this figure is available at https://blobtoolkit.genomehubs.org/view/Charanyca%20ferruginea/dataset/CANAIE01/blob.

**Figure 4.**
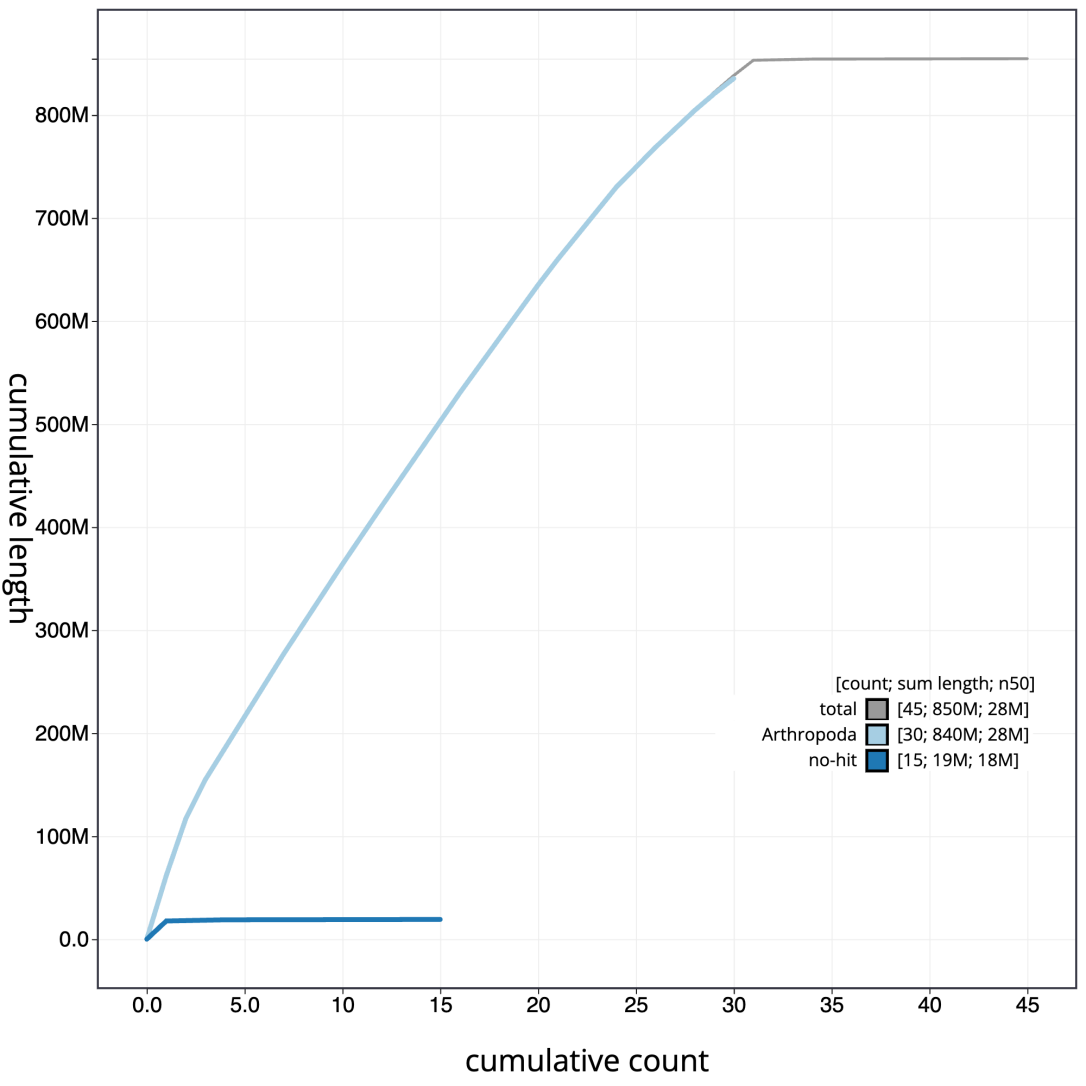
Genome assembly of *Charanyca ferruginea*, ilChaFerr1.1: BlobToolKit cumulative sequence plot. The grey line shows cumulative length for all scaffolds. Coloured lines show cumulative lengths of scaffolds assigned to each phylum using the buscogenes taxrule. An interactive version of this figure is available at https://blobtoolkit.genomehubs.org/view/Charanyca%20ferruginea/dataset/CANAIE01/cumulative.

**Figure 5.**
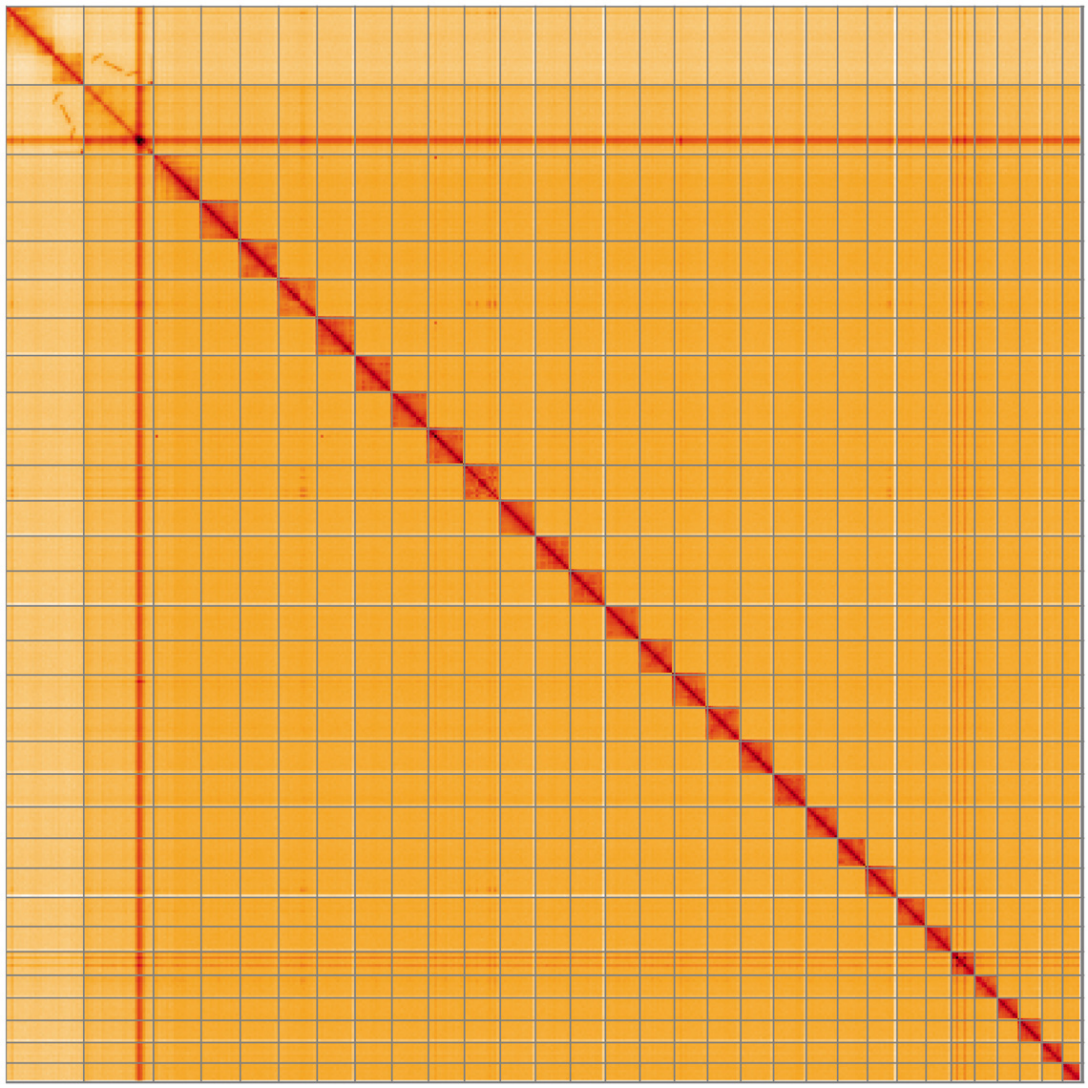
Genome assembly of *Charanyca ferruginea*, ilChaFerr1.1: Hi-C contact map of the ilChaFerr1.1 assembly, visualised using HiGlass. Chromosomes are shown in order of size from left to right and top to bottom. An interactive version of this figure may be viewed at https://genome-note-higlass.tol.sanger.ac.uk/l/?d=Rr_u06gvQG65l-FLLKvYow.

The final assembly has a total length of 854.6 Mb in 44 sequence scaffolds with a scaffold N50 of 27.8 Mb ( Table 1). The snailplot in Figure 2 provides a summary of the assembly statistics. The distribution of assembly scaffolds on GC proportion and coverage is shown in Figure 3, and the cumulative assembly plot in Figure 4 shows curves for subsets of scaffolds assigned to different phyla. Most (99.97%) of the assembly sequence was assigned to 31 chromosomal-level scaffolds, representing 29 autosomes and the Z and W sex chromosomes. Chromosome-scale scaffolds confirmed by the Hi-C data are named in order of size ( Figure 5; Table 2). The Hi-C data indicate some collinearity between the Z and W chromosomes. While not fully phased, the assembly deposited is of one haplotype. Contigs corresponding to the second haplotype have also been deposited. The mitochondrial genome was also assembled and can be found as a contig within the multifasta file of the genome submission.

**Table 1.** Genome data for *Charanyca ferruginea*, ilChaFerr1.1.

Project accession data		
Assembly identifier	ilChaFerr1.1	
Species	*Charanyca ferruginea*	
Specimen	ilChaFerr1	
NCBI taxonomy ID	987475	
BioProject	PRJEB55737	
BioSample ID	SAMEA7631559	
Isolate information	ilChaFerr1, female: thorax (DNA sequencing), abdomen (Hi-C scaffolding)	
Assembly metrics *		*Benchmark*
Consensus quality (QV)	65.3	*≥ 50*
*k*-mer completeness	100%	*≥ 95%*
BUSCO **	C:98.9%[S:95.9%,D:3.0%],F:0.2%,M:0.9%, n:5,286	*C ≥ 95%*
Percentage of assembly mapped to chromosomes	99.97%	*≥ 95%*
Sex chromosomes	Z and W chromosomes	*localised homologous pairs*
Organelles	Mitochondrial genome assembled	*complete single alleles*
Raw data accessions		
PacificBiosciences SEQUEL IIe	ERR10168724, ERR10168725, ERR10168726	
Hi-C Illumina	ERR10149556	
Genome assembly		
Assembly accession	GCA_947361185.1	
*Accession of alternate haplotype*	GCA_947366815.1	
Span (Mb)	854.6	
Number of contigs	159	
Contig N50 length (Mb)	19.4	
Number of scaffolds	44	
Scaffold N50 length (Mb)	27.8	
Longest scaffold (Mb)	62.0	
Genome annotation		
Number of protein-coding genes	23,126	
Number of gene transcripts	23,326	

* Assembly metric benchmarks are adapted from column VGP-2020 of “Table 1: Proposed standards and metrics for defining genome assembly quality” from ( Rhie *et al.*, 2021). ** BUSCO scores based on the lepidoptera_odb10 BUSCO set using v5.3.2. C = complete [S = single copy, D = duplicated], F = fragmented, M = missing, n = number of orthologues in comparison. A full set of BUSCO scores is available at https://blobtoolkit.genomehubs.org/view/ilChaFerr1.1/dataset/CANAIE01/busco.

**Table 2.** Chromosomal pseudomolecules in the genome assembly of *Charanyca ferruginea*, ilChaFerr1.

INSDC accession	Chromosome	Length (Mb)	GC%
OX376057.1	1	37.84	37.5
OX376058.1	2	30.99	37.5
OX376059.1	3	30.5	37.5
OX376060.1	4	30.45	37.5
OX376061.1	5	29.95	37.0
OX376062.1	6	29.21	37.0
OX376063.1	7	29.06	37.5
OX376064.1	8	28.7	37.5
OX376065.1	9	28.26	37.5
OX376066.1	10	27.93	37.5
OX376067.1	11	27.76	37.5
OX376068.1	12	27.73	37.0
OX376069.1	13	27.46	37.5
OX376070.1	14	27.23	37.5
OX376072.1	16	26.34	37.5
OX376071.1	15	26.34	37.5
OX376073.1	17	26.08	37.0
OX376074.1	18	25.96	37.5
OX376075.1	19	24.76	37.5
OX376076.1	20	23.74	37.5
OX376077.1	21	23.41	37.5
OX376078.1	22	23.11	37.5
OX376079.1	23	19.9	37.5
OX376080.1	24	18.65	39.0
OX376081.1	25	18.04	38.0
OX376082.1	26	17.74	37.5
OX376083.1	27	17.73	37.5
OX376084.1	28	16.25	38.0
OX376085.1	29	14.77	38.0
OX376056.1	W	55.28	38.5
OX376055.1	Z	61.98	37.0
OX376086.1	MT	0.02	18.5

The estimated Quality Value (QV) of the final assembly is 65.3 with *k*-mer completeness of 100%, and the assembly has a BUSCO v5.3.2 completeness of 98.9% (single = 95.9%, duplicated = 3.0%), using the lepidoptera_odb10 reference set ( *n* = 5,286).

Metadata for specimens, spectral estimates, sequencing runs, contaminants and pre-curation assembly statistics can be found at https://links.tol.sanger.ac.uk/species/987475.

## Genome annotation report

The *Charanyca ferruginea* genome assembly (GCA_947361185.1) was annotated using the Ensembl rapid annotation pipeline ( Table 1; https://rapid.ensembl.org/Charanyca_ferruginea_GCA_947361185.1/Info/Index). The resulting annotation includes 23,326 transcribed mRNAs from 23,126 protein-coding genes.

## Methods

### Sample acquisition and nucleic acid extraction

A female *Charanyca ferruginea* (specimen ID Ox000445, individual ilChaFerr1) was collected from Wytham Woods, Oxfordshire (biological vice-county Berkshire), UK (latitude 51.77, longitude –1.34) on 2020-06-13, using a light trap. The specimen was collected and identified by Douglas Boyes (University of Oxford) and was snap-frozen on dry ice.

DNA was extracted at the Tree of Life laboratory, Wellcome Sanger Institute (WSI). The ilChaFerr1 sample was weighed and dissected on dry ice with tissue set aside for Hi-C sequencing. Abdomen tissue was disrupted using a Nippi Powermasher fitted with a BioMasher pestle *]*. High molecular weight (HMW) DNA was extracted using the Qiagen MagAttract HMW DNA extraction kit. Low molecular weight DNA was removed from a 20 ng aliquot of extracted DNA using the 0.8X AMpure XP purification kit prior to 10X Chromium sequencing; a minimum of 50 ng DNA was submitted for 10X sequencing. HMW DNA was sheared into an average fragment size of 12–20 kb in a Megaruptor 3 system with speed setting 30. Sheared DNA was purified by solid-phase reversible immobilisation using AMPure PB beads with a 1.8X ratio of beads to sample to remove the shorter fragments and concentrate the DNA sample. The concentration of the sheared and purified DNA was assessed using a Nanodrop spectrophotometer and Qubit Fluorometer and Qubit dsDNA High Sensitivity Assay kit. Fragment size distribution was evaluated by running the sample on the FemtoPulse system.

### Sequencing

Pacific Biosciences HiFi circular consensus DNA sequencing libraries were constructed according to the manufacturers’ instructions. DNA sequencing was performed by the Scientific Operations core at the WSI on a Pacific Biosciences SEQUEL II (HiFi) instrument. Hi-C data were also generated from thorax tissue of ilChaFerr1 using the Arima2 kit and sequenced on the Illumina NovaSeq 6000 instrument.

### Genome assembly, curation and evaluation

Assembly was carried out with Hifiasm ( Cheng *et al.*, 2021) and haplotypic duplication was identified and removed with purge_dups ( Guan *et al.*, 2020). The assembly was then scaffolded with Hi-C data ( Rao *et al.*, 2014) using YaHS ( Zhou *et al.*, 2023). The assembly was checked for contamination and corrected as described previously ( Howe *et al.*, 2021). Manual curation was performed using HiGlass ( Kerpedjiev *et al.*, 2018) and Pretext ( Harry, 2022). The mitochondrial genome was assembled using MitoHiFi ( Uliano-Silva *et al.*, 2022), which runs MitoFinder ( Allio *et al.*, 2020) or MITOS ( Bernt *et al.*, 2013) and uses these annotations to select the final mitochondrial contig and to ensure the general quality of the sequence.

A Hi-C map for the final assembly was produced using bwa-mem2 ( Vasimuddin *et al.*, 2019) in the Cooler file format ( Abdennur & Mirny, 2020). To assess the assembly metrics, the *k*-mer completeness and QV consensus quality values were calculated in Merqury ( Rhie *et al.*, 2020). This work was done using Nextflow ( Di Tommaso *et al.*, 2017) DSL2 pipelines “sanger-tol/readmapping” ( Surana *et al.*, 2023a) and “sanger-tol/genomenote” ( Surana *et al.*, 2023b). The genome was analysed within the BlobToolKit environment ( Challis *et al.*, 2020) and BUSCO scores ( Manni *et al.*, 2021; Simão *et al.*, 2015) were calculated.

Table 3 contains a list of relevant software tool versions and sources.

**Table 3.** Software tools: versions and sources.

Software tool	Version	Source
BlobToolKit	4.0.7	https://github.com/blobtoolkit/blobtoolkit
BUSCO	5.3.2	https://gitlab.com/ezlab/busco
Hifiasm	0.16.1-r375	https://github.com/chhylp123/hifiasm
HiGlass	1.11.6	https://github.com/higlass/higlass
Merqury	MerquryFK	https://github.com/thegenemyers/MERQURY.FK
MitoHiFi	2	https://github.com/marcelauliano/MitoHiFi
PretextView	0.2	https://github.com/wtsi-hpag/PretextView
purge_dups	1.2.3	https://github.com/dfguan/purge_dups
sanger-tol/genomenote	v1.0	https://github.com/sanger-tol/genomenote
sanger-tol/readmapping	1.1.0	https://github.com/sanger-tol/readmapping/tree/1.1.0
YaHS	yahs-1.1.91eebc2	https://github.com/c-zhou/yahs

### Genome annotation

The BRAKER2 pipeline ( Brůna *et al.*, 2021) was used in the default protein mode to generate annotation for the *Charanyca ferruginea* assembly (GCA_947361185.1) in Ensembl Rapid Release.

### Wellcome Sanger Institute – Legal and Governance

The materials that have contributed to this genome note have been supplied by a Darwin Tree of Life Partner. The submission of materials by a Darwin Tree of Life Partner is subject to the **‘Darwin Tree of Life Project Sampling Code of Practice’**, which can be found in full on the Darwin Tree of Life website here. By agreeing with and signing up to the Sampling Code of Practice, the Darwin Tree of Life Partner agrees they will meet the legal and ethical requirements and standards set out within this document in respect of all samples acquired for, and supplied to, the Darwin Tree of Life Project. 

Further, the Wellcome Sanger Institute employs a process whereby due diligence is carried out proportionate to the nature of the materials themselves, and the circumstances under which they have been/are to be collected and provided for use. The purpose of this is to address and mitigate any potential legal and/or ethical implications of receipt and use of the materials as part of the research project, and to ensure that in doing so we align with best practice wherever possible. The overarching areas of consideration are:

•   Ethical review of provenance and sourcing of the material

•   Legality of collection, transfer and use (national and international)

Each transfer of samples is further undertaken according to a Research Collaboration Agreement or Material Transfer Agreement entered into by the Darwin Tree of Life Partner, Genome Research Limited (operating as the Wellcome Sanger Institute), and in some circumstances other Darwin Tree of Life collaborators.

## Data Availability

European Nucleotide Archive: *Charanyca ferruginea* (brown rustic). Accession number PRJEB55737; https://identifiers.org/ena.embl/PRJEB55737. ( Wellcome Sanger Institute, 2022) The genome sequence is released openly for reuse. The *Charanyca ferruginea* genome sequencing initiative is part of the Darwin Tree of Life (DToL) project. All raw sequence data and the assembly have been deposited in INSDC databases. Raw data and assembly accession identifiers are reported in Table 1.

## Author information

Members of the University of Oxford and Wytham Woods Genome Acquisition Lab are listed here: https://doi.org/10.5281/zenodo.4789928.

Members of the Darwin Tree of Life Barcoding collective are listed here: https://doi.org/10.5281/zenodo.4893703.

Members of the Wellcome Sanger Institute Tree of Life programme are listed here: https://doi.org/10.5281/zenodo.4783585.

Members of Wellcome Sanger Institute Scientific Operations: DNA Pipelines collective are listed here: https://doi.org/10.5281/zenodo.4790455.

Members of the Tree of Life Core Informatics collective are listed here: https://doi.org/10.5281/zenodo.5013541.

Members of the Darwin Tree of Life Consortium are listed here: https://doi.org/10.5281/zenodo.4783558.
