# Supplementary material for: The genome sequence of the Brown Rustic, Charanyca ferruginea (Esper, 1785)
Source: Wellcome Open Res. 2023 Nov 23;8:547. [Version 1] doi: 10.12688/wellcomeopenres.20137.1 (PMC11316178; doi:10.12688/wellcomeopenres.20137.1)
Supplement: Peer review history

# Reviewer report: Reviewer response for version 1

**DOI:** 10.21956/wellcomeopenres.22299.r90363

**Published:** 2024-08-12

**Authors:** Nicola Nadeau

**Affiliations:** 1 The University of Sheffield, Sheffield, UK

This paper reports a genome assembly of the Brown Rustic moth. It is assembled to chromosome level. It will be particularly useful to evolutionary studies that the Z and W have been fully assembled, since a female individual was sequenced. It could perhaps be clarified if any RNA sequence data was used in the annotation. It seems not, which is one limitation of the annotation that could be acknowledged.

Are sufficient details of methods and materials provided to allow replication by others?

Yes

Is the rationale for creating the dataset(s) clearly described?

Yes

Are the datasets clearly presented in a useable and accessible format?

Yes

Are the protocols appropriate and is the work technically sound?

Yes

Reviewer Expertise:

Evolutionary biology, genomics, lepidoptera

I confirm that I have read this submission and believe that I have an appropriate level of expertise to confirm that it is of an acceptable scientific standard.

# Reviewer report: Reviewer response for version 1

**DOI:** 10.21956/wellcomeopenres.22299.r90366

**Published:** 2024-08-09

**Authors:** Thomas Brown

**Affiliations:** 1 Berlin Center for Genomics in Biodiversity Research, Berlin, Berlin, Germany; 2 Leibniz IZW (Berlin) & ERGA/BGP, Leibniz, Germany

Boyes et al. present a chromosome-scale genome assembly of the Brown Rustic, a moth from the British Isles. The genome is of high quality and the methodology describing the sampling and DNA extraction are well described. There are a few issues I wish to highlight to ensure that the process is truly transparent and can be interpreted fully by the readers.

 The introduction to the species itself is very nicely written and interesting. It would benefit from an explanation of what having a genome assembly will bring to the study of this species.

 In the "sequencing" section of the Methods it is not stated how the 10X library was sequenced and indeed what this data was used for, as it is not listed in Table 1, despite being included in the sequencing BioProject (I am having to guess a bit, but it looks like this is the following runs: ERR10149553, ERR10149554, ERR10149555) and apparently one of these experiments was used to generate Figure 3.

**Figure 3:**

 It is not clear from the figure legend what the y axis corresponds to. In fact only by searching for the accession number listed can one find out that this corresponds to a WGS dataset which is not listed within the rest of the article. Given that in the "Genome sequence report" section it is stated that 46-fold HiFi coverage was generated, I feel it would make more sense to generate the blobplot using this dataset, rather than the one used, which looks to only be at 20-fold coverage.

**Table 1:**

 The stated 100% kmer completeness must correspond to the value obtained when combining the primary and alternate assemblies together. Is this also true for the QV value? The BUSCO and % assigned to chromosome values must be from the primary assembly only. I find this a little bit confusing and has to potential to mislead people and should be made much clearer.

 As I have brought up in a previous Data Note from the same consortium, I find the reporting of the genome annotation incomplete. Stating the number of genes and transcripts alone does not give the reader sufficient information to establish the quality and usefulness of the annotation alongside the genome assembly. As a minimum, I would like to see the BUSCO completeness scores as well as OMArk completeness and consistency scores of the annotated sequences. This should also be coupled with a report of the number of exons per gene on average, the number of single-exon genes.

 The methods section for the genome annotation is not informative or complete. While Braker2 is listed as the annotation tool, there is no indication of whether the genome was repeat masked (and how) and what supporting evidences were used to generate the gene model predictions - i.e which set of protein sequences were used in the "default protein mode" as is stated in the text.

Are sufficient details of methods and materials provided to allow replication by others?

Partly

Is the rationale for creating the dataset(s) clearly described?

Partly

Are the datasets clearly presented in a useable and accessible format?

Yes

Are the protocols appropriate and is the work technically sound?

Yes

Reviewer Expertise:

Genome assembly, genome annotation

I confirm that I have read this submission and believe that I have an appropriate level of expertise to confirm that it is of an acceptable scientific standard, however I have significant reservations, as outlined above.

# Reviewer report: Reviewer response for version 1

**DOI:** 10.21956/wellcomeopenres.22299.r90360

**Published:** 2024-08-09

**Authors:** Doga Cedden

**Affiliations:** 1 Department of Evolutionary Developmental Genetics, Johann-Friedrich-Blumenbach Institute, Göttingen, Germany

Boyes & Hutchinson report a high-quality genome assembly from *Charanyca ferruginea. *The authors provided adequate background information regarding the *C. ferruginea*. The methodology is clearly described and appropriate. Sufficient quality control is provided regarding the genome assembly. The provided data depository contains the necessary data. 

 I only have one concern regarding the mitochondrial genome assembly. The reported GC% is 18.5 for the mitochondrial genome assembly. This seems low, but similar or even lower mitochondrial GC% contents are also observed mitochondrial genome GC% is observed in other insects. This could be at least briefly mentioned in the text for unfamiliar readers.

Are sufficient details of methods and materials provided to allow replication by others?

Yes

Is the rationale for creating the dataset(s) clearly described?

Yes

Are the datasets clearly presented in a useable and accessible format?

Yes

Are the protocols appropriate and is the work technically sound?

Yes

Reviewer Expertise:

I work on coleopteran pests mainly using RNA-seq and RNA interference methods.

I confirm that I have read this submission and believe that I have an appropriate level of expertise to confirm that it is of an acceptable scientific standard.

# Reviewer report: Reviewer response for version 1

**DOI:** 10.21956/wellcomeopenres.22299.r90369

**Published:** 2024-08-09

**Authors:** Lapo Ragionieri

**Affiliations:** 1 Libera Universita di Bolzano (Ringgold ID: 18956), Bolzano, Trentino-Alto Adige/South Tyrol, Italy; 2 University of Cologne, Cologne, Germany

The data note " The genome sequence of the Brown Rustic, *Charanyca ferruginea*" by Douglas Boyes and Finley Hutchinson describes the sequencing and genome assembly of *Charanyca ferruginea*. The authors utilized long-read sequencing technology (PacBio) to produce an initial assembly that was subsequently scaffolded using Hi-C, resulting in a genome assembly at the chromosome level with nearly all scaffolds assigned to chromosomes. The genome annotation includes 23,326 transcribed mRNAs. The presentation of the results are clear, but the aims should be more clear why this species was selected and I would also expect to see the results of genome masking *.*

**Below are some more specific comments and suggestions:**

- **Keywords:** The keywords need to be different from the title otherwise are useless.
- **Species identification: **the author should cite the literature used.
- **Mitochondrial genome**: I strongly recommend submitting the genome separated and with an independent accession number. Moreover, the authors described the methods but did not provide any information about the mitochondrial genome annotation (coding genes, rRNAs, and tRNAs). I would expect that the authors submit the annotations obtained by MITOS separately.
- **Annotation**: The authors should identify and quantify the transposable elements.
- **Command lines**: It is common practice to include the command lines used with all software. I believe this information can be very useful for the readers.

Are sufficient details of methods and materials provided to allow replication by others?

Partly

Is the rationale for creating the dataset(s) clearly described?

Partly

Are the datasets clearly presented in a useable and accessible format?

Yes

Are the protocols appropriate and is the work technically sound?

Yes

Reviewer Expertise:

Genomic, transcriptomic and proteomic

I confirm that I have read this submission and believe that I have an appropriate level of expertise to confirm that it is of an acceptable scientific standard, however I have significant reservations, as outlined above.

# Reviewer report: Reviewer response for version 1

**DOI:** 10.21956/wellcomeopenres.22299.r90364

**Published:** 2024-08-06

**Authors:** Arun Arumugaperumal

**Affiliations:** 1 Department of Biotechnology, Rajalakshmi Engineering College, Chennai, Tamilnadu, India

This is the first report of the whole genome of the insect *Charanyca ferruginea*. The insect goes by the common name Brown Rustic. The genome assembly reported in this data article is of size 854.6 Mbp. The scaffold consisted of 31 chromosomes and a mitogenome. The gene annotation with Ensembl has identified 23,126 protein coding genes. 

 I have no knowledge of the species taxonomy reported here. So I could not comment on it. The photograph provided as figure 1 is of good quality. The authors clearly explained the morphology features and appearance of the insect. It would have been better if they had shown a photo of live insect along with the specimen sample. 

 The authors have done 46 fold coverage sequencing in long read sequencing technology. The contig N50 value was sufficiently high. BUSCO analysis gives an idea of completeness of the genome sequence. As the results indicate 98% completeness we can assume the genome sequence is a near complete one.

 Not much research work was done using this insect as evident from the literature search using the insect name. The genome sequence reported here with might form basis for designing molecular biology works in the future. 

 There is an unwanted symbol trailing the words 'BioMasher pestle].' in Methods section.

Are sufficient details of methods and materials provided to allow replication by others?

Yes

Is the rationale for creating the dataset(s) clearly described?

Yes

Are the datasets clearly presented in a useable and accessible format?

Yes

Are the protocols appropriate and is the work technically sound?

Yes

Reviewer Expertise:

Bioinformatics, Genomics

I confirm that I have read this submission and believe that I have an appropriate level of expertise to confirm that it is of an acceptable scientific standard.
